# Effects of Modified Chicken Manure Biochar-Based *Bacillus velezensis* Agent on Vegetable Growth, Saline–Alkali Soil Properties, and Microbial Communities

**DOI:** 10.3390/plants15192987

**Published:** 2026-09-30

**Authors:** Siyi Zhang, Lijuan Sun, Shuangxi Li, Xianpu Zhu, Haiyun Zhang, Yue Zhang, Weiguang Lv, Tinglin Zhang, Chenglong Xu, Hanlin Zhang, Juanqin Zhang, Naling Bai

**Affiliations:** 1 Eco-Environmental Protection Research Institute, Shanghai Academy of Agricultural Sciences, Shanghai 201403, China; 18235645196@163.com (S.Z.); sunlijuan@saas.sh.cn (L.S.); lishuangxi@saas.sh.cn (S.L.); zhuxianpu@saas.sh.cn (X.Z.); zhanghaiyun@saas.sh.cn (H.Z.); zhangyue@saas.sh.cn (Y.Z.); lvweiguang@saas.sh.cn (W.L.); xuchenglong@saas.sh.cn (C.X.); zhanghanlin@saas.sh.cn (H.Z.); 2 Key Laboratory of Low-Carbon Green Agriculture in Southeastern China, Ministry of Agriculture and Rural Affairs, Shanghai 201403, China; 3 Shanghai Agri-Environmental Protection Monitoring Station, Shanghai 201403, China; 4 Shanghai Engineering Research Center of Low-Carbon Agriculture, Shanghai 201415, China; 5 Shanghai Qingpu Agricultural Technology Extension Service Center, Shanghai 201799, China; 15900966070@163.com; 6 National Agricultural Experimental Station for Agricultural Environment, Fengxian, Shanghai 201415, China

**Keywords:** saline–alkali soil, biochar-based agent, acid modification, pakchoi production, soil improvement, microbial community

## Abstract

Soil salinization limits sustainable agriculture. This study used a citric acid–FeSO_4_ method to create modified chicken manure biochar (MCMB) as a carrier for the plant growth-promoting *Bacillus velezensis* SS-20, forming a biochar-based microbial agent (JS). A pot experiment included four treatments: CK (control), SS (SS-20 only), JF (MCMB only), and JS (biochar-based agent). The JS treatment significantly improved pakchoi plant height and aboveground fresh weight by 26.33% and 76.62%, respectively, compared to CK. It also lowered soil pH, electrical conductivity (EC), and salt content, while enhancing soil catalase activity by 8.83%. The ameliorative effects of SS and JF were less pronounced than those of JS. The contribution of biochar and SS-20 to the improvement of the soil quality index suggests additive effects with minimal interaction. Redundancy analysis identified EC and total potassium as the key environmental variables influencing microbial community structure at the phylum and genus levels, respectively. JS influenced the relative abundances of candidate lineages, such as *Myxococcota*, *AG11*, and *Gemmatirosa*, as well as functional genes associated with energy metabolism and cell motility. In summary, JS enhanced soil properties, modulated differentially abundant taxa and their metabolic potential, and consequently facilitated pakchoi production in saline–alkali soil. This study offers practical insights for the ecological management of saline–alkali farmland.

## 1. Introduction

Soil salinization is a major abiotic stress threatening global agricultural sustainability [1]. Saline–alkali land is estimated at 1.0 × 10^9^ hm^2^ worldwide, of which approximately 99.13 million hm^2^ is located in China [2]. Salt and pH stresses suppress soil biological activity and diversity, destabilize soil ecosystems, and ultimately endanger food security [3,4]. Developing practical, low-cost amendments for these soils is therefore a pressing priority.

Conventional remediation strategies, such as irrigation engineering, chemical amendments, and agronomic improvements, are frequently economically burdensome and provide only temporary effects [5,6]. Biochar has emerged as a more environmentally sustainable alternative. Biochar is produced through the pyrolysis of biomass under oxygen-limited conditions, resulting in a material with a high specific surface area, elevated cation exchange capacity, and a stable aromatic carbon structure, which together enhance soil porosity and water retention [7]. A recent meta-analysis encompassing 137 studies showed that biochar reduces soil sodium adsorption ratio and exchangeable sodium percentage (ESP) by approximately 30%, accompanied with significant increments in soil organic carbon and crop productivity [7]. Nevertheless, the efficacy of biochar is highly contingent upon the type of feedstock used and the application rate. Many plant-based biochars release insufficient nutrients to support crops throughout their entire growth cycle [8], whereas high-dose applications can release alkaline ash, increase soil pH and electrical conductivity (EC), and exacerbate saline–alkali stress [9,10]. Overall, the inherent strong alkalinity and high mineral salt content of raw biochar suggest that excessive application may be detrimental to plant health.

Functional microorganisms provide a complementary biological strategy. Plant growth-promoting bacteria can secrete phytohormones, activate soil nutrients, and induce systemic resistance [11]. For example, members of *Pseudomonadaceae* and *Burkholderiaceae B* produce extracellular polysaccharides that accelerate soil aggregation [12], while *Bacillus amyloliquefaciens* SQR9 alleviates sodium phytotoxicity by regulating ion transporter genes (e.g., *NHX1*, *H-PPase*, and *HKT1*) [13]. Direct field application of such microbes faces bottlenecks, including poor colonization, low survival rate, and weak competition against indigenous microorganisms [14]. The high osmotic pressure and ionic toxicity of saline–alkali soils rapidly inactivate exogenous strains, resulting in unstable field performance [3].

Immobilizing microorganisms on biochar may overcome these mutual limitations. Its porous structure shelters cells and prolongs their survival, while surface functional groups retain them through electrostatic adsorption [15]; the microbes, in turn, remodel the biochar surface and drive nutrient transformation. A field trial in Heilongjiang showed that a biochar–microbial composite lowered soil pH by 2.16 units and ESP by 14.64% [16]. Yet most studies have used plant-residue biochars, which provide habitat but little inherent nutrition [8]. Livestock manure biochars remain comparatively unexplored. Among them, chicken manure biochar stands out for its abundant mineral nutrients and organic nitrogen, giving it dual value as both a soil amendment and a microbial carrier [17]. One obstacle remains that chicken manure biochar is typically strongly alkaline (pH 9–13), far above the optimum (pH 6–8) for most functional microbes, so acid modification is a prerequisite [8]. Here we adopted a mild organic acid strategy. FeSO_4_ releases H^+^ through hydrolysis and thus moderates pH without severe structural damage. Citric acid, a natural intermediate of the tricarboxylic acid cycle, supplies sustained acidity and enriches the surface with carboxyl and hydroxyl groups [18]. These oxygen-containing groups enhance electrostatic attraction and hydrogen bonding with microbial cell walls. Together, the citric acid–FeSO_4_ treatment achieves pH neutralization and surface functionalization in one step, producing a nutrient-rich carrier chemically compatible with microbial immobilization.

In this study, we prepared a biochar-based microbial agent by immobilizing *Bacillus velezensis* SS-20, a plant growth-promoting bacterium [11], onto acid-modified chicken manure biochar (MCMB). Our hypothesis posited that this modified carrier would deliver essential minerals to saline–alkali soils while maintaining a pH-neutral environment conducive to the sustained activity of the immobilized bacteria. We assessed the agent’s characteristics and investigated its impact on vegetable productivity, soil physicochemical properties, enzyme activities, and microbial community structure through pot experiments. Importantly, we utilized an integrated evaluation approach, combining a soil quality index (SQI) with contribution rate analysis, to determine whether the combined effects of biochar and microbial inoculants were synergistic, additive, or antagonistic in saline–alkali environments. This approach facilitated a comprehensive and agriculturally relevant assessment of soil health improvement. Our findings elucidate the mechanisms by which biochar-based microbial amendments ameliorate saline–alkali soils and provide a practical foundation for sustainable agricultural practices.

## 2. Results

### 2.1. Characteristics of Biochar-Based Agent

**Figure 1:**
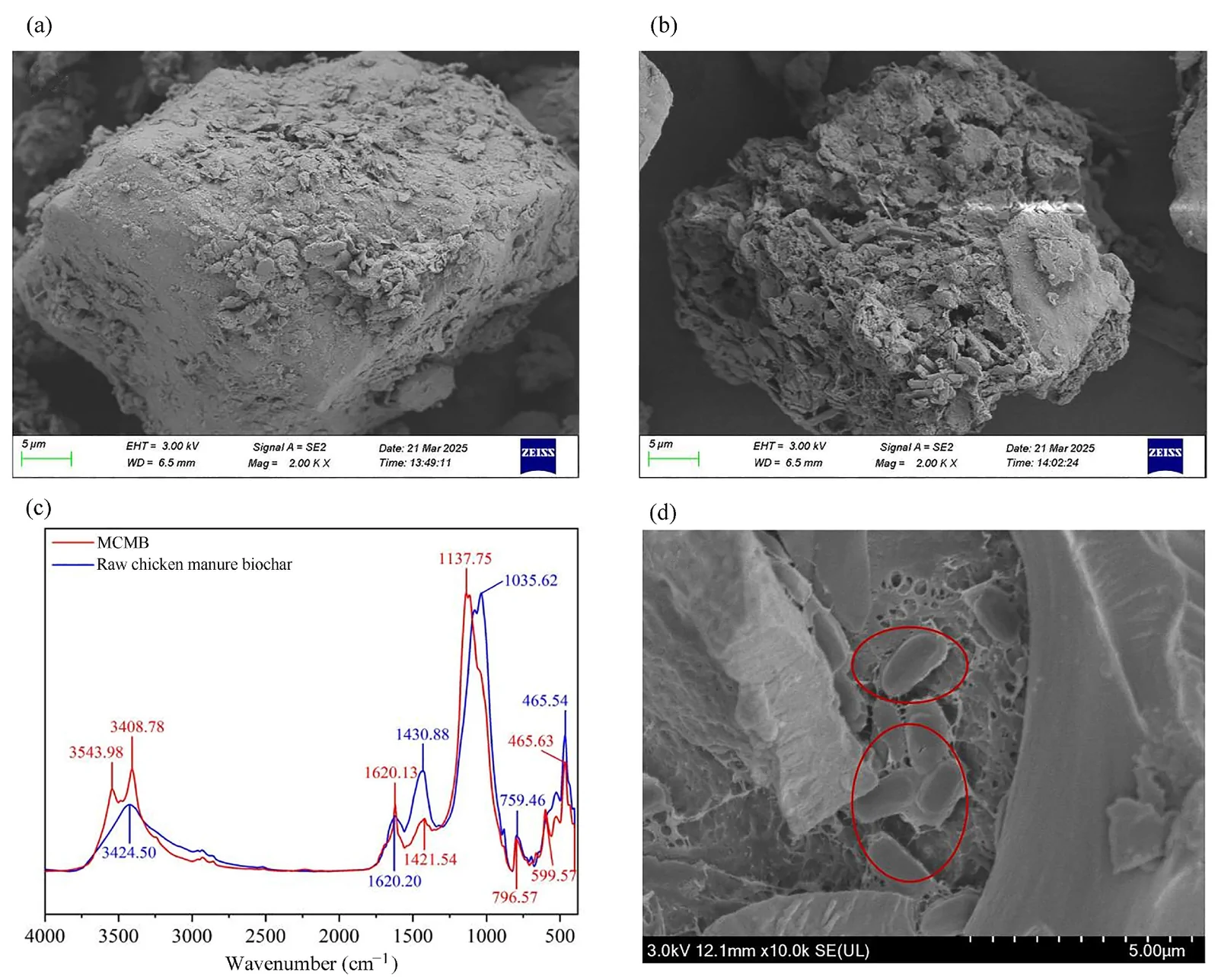
SEM images of chicken manure biochar before (**a**) and after (**b**) acid modification, FTIR spectra profiles (**c**), and cryo-SEM images of biochar-based agents (**d**). MCMB refers to chicken manure biochar with acid modification; red circles indicate immobilized microbial cells on MCMB.

Citric acid–FeSO_4_ treatment modified the surface pore structure of the MCMB (Figure 1a,b). SEM images revealed a rougher surface and altered pore morphology, and BET analysis confirmed increases of 72.80%, 1007.01%, and 4.13% in specific surface area, micropore volume, and porosity, respectively (Table S1). Elemental analysis revealed increases in the H, N, and S contents by 0.23, 0.31, and 2.17 percentage points, respectively, whereas the K, P, Na, and Mg contents decreased by 1.40, 1.33, 0.274, and 0.77 percentage points, respectively (Table S2). The FTIR spectra (Figure 1c) revealed –OH stretching bands at 3200–3450 cm^−1^ in both samples, along with aromatic C=C stretching (1620 cm^−1^), C–H bending (1430 and 795 cm^−1^), and C–O stretching (1035–1138 cm^−1^) peaks. The band near 465 cm^−1^ (Si–O–Si bending) weakened after modification. Two new peaks at 3543.98 cm^−1^ and 599.57 cm^−1^ in MCMB spectrum were assigned to free –OH groups and inorganic mineral lattice vibrations, respectively. Abundant SS-20 cells were observed on the MCMB surface, with some cells anchored within the pores (Figure 1d).

### 2.2. Effects of Biochar-Based Agent on Vegetable Indices

Compared with CK, the external amendments (SS, JF, and JS) increased the aboveground and belowground fresh and dry weights of pakchoi, as shown in Table 1, with the JS treatment demonstrating the most pronounced effect (*p* < 0.05). The plant height in the JS group was significantly greater than that in CK and JF, with increases of 26.33% and 20.45%, respectively (*p* < 0.05). The growth traits in the SS and JF groups exhibited only moderate improvements compared to the CK group. Bacterial inoculation significantly increased plant height (*p* = 0.013), while both the biochar and bacteria individually increased vegetable biomass, including aboveground fresh weight (*p* = 0.005–0.016), underground fresh weight (*p* = 0.003–0.009), aboveground dry weight (*p* = 0.004–0.013), and underground dry weight (*p* = 0.025–0.026). There was a significant interaction between biochar and bacteria on aboveground fresh weight (*p* = 0.045).

Although chlorophyll content, an indicator of photosynthetic efficiency and vegetable quality [19], tended to be higher in all three amendment treatments compared to CK, the differences were not statistically significant (*p* > 0.05), with increases ranging from 6.06% to 12.12%. Root activity was highest in the JS group (0.572 μg/min/g) and was significantly greater than that observed in the SS group (*p* < 0.05; Table 2). Leaf malondialdehyde (MDA) and proline (Pro) contents and superoxide dismutase (SOD) and peroxidase (POD) activities reflect plant stress tolerance [20]. These parameters did not differ significantly among the four treatments (*p* > 0.05; Table 2). The JS treatment resulted in the best performance, while the physiological indices (chlorophyll content, root activity, and antioxidant indices) did not significantly differ according to one-way ANOVA and two-way ANOVA. Under the salinity–alkalinity stress intensity in this study, the exogenous amendments did not markedly alter the antioxidant defense system of pakchoi.

### 2.3. Effects of Bio-Based Agent on Soil Properties

In comparison to CK, the SS, JF, and JS treatments significantly decreased the soil pH by 0.33, 0.35, and 0.27 units, respectively (*p* < 0.05). The JS treatment notably decreased the soil salt content by 37.97% (*p* < 0.05), with EC exhibiting a similar pattern (Table 3). Among the treatment groups, the SS group demonstrated significantly elevated TP levels, with increases ranging from 0.003 to 0.026 percentage points (*p* < 0.05). SS, JF, and JS tended to elevate the SOM content (*p* > 0.05), with JS showing the greatest increase of 0.71 percentage points. No significant differences were detected in the SOM, TN or TK contents across treatments.

Soil enzyme activity was responsive to the amendments, with S-CAT activity in the SS and JS treatments (66.29 and 66.91 μmol/d/g, respectively) being significantly higher than in the CK and JF groups (*p* < 0.05), representing increases of 7.82% and 8.83% compared to CK. Although JF and JS showed a tendency to enhance S-UE activity, and the SS and JS treatments tended to increase S-SC activity, these changes were not statistically significant.

Biochar application significantly reduced soil pH, EC, and salt content (*p* = 0.003, *p* = 0.007, *p* < 0.001). In contrast, bacterial inoculation led to a significant increase in S-CAT activity (*p* = 0.002) and TP content (*p* = 0.007), while also contributing to a decrease in soil pH (*p* = 0.008). Notably, significant interactions between biochar application and bacterial inoculation were detected for pH, EC, and TP content (*p* < 0.001, *p* = 0.010, *p* = 0.050, respectively). However, no significant interaction effects were observed for SOM, TN, TK contents, or soil enzyme activities.

### 2.4. Soil Quality Index and Contribution Rate

Compared with CK treatment, JS resulted in the highest soil quality index (SQI = 0.614 ± 0.077), representing a 96.23% improvement, and was markedly superior to either the single SS-20 inoculant application or the biochar amendment (Table 4). The Shapley value revealed that, compared with the SS-20 inoculant (44.33%, φ = 0.134), the biochar contributed more (55.67%, φ = 0.168) to the increase in the SQI, with a synergy value of merely +0.004, indicating an approximately additive interaction.

### 2.5. Variations in Soil Microbial Community Structure

#### 2.5.1. The α and β Diversity Analyses

**Figure 2:**
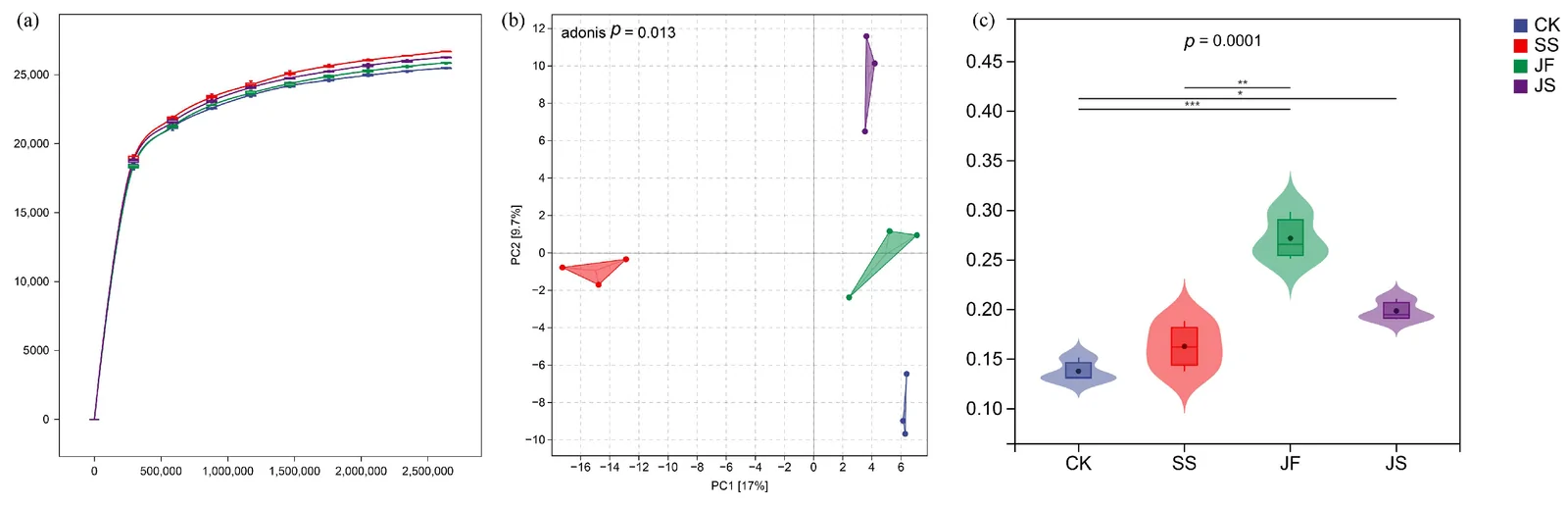
Rarefaction curve (**a**); PLS-DA of β diversity at the order level (F = 2.03, R^2^ = 0.43, *p* = 0.023; 999 permutations) (**b**); Bray–Curtis dissimilarity at the species level, with significant differences assessed by the Kruskal–Wallis test followed by Dunn’s post hoc test (**c**). * *p* < 0.05; ** *p* < 0.01; *** *p* < 0.001. CK: blank control; SS: SS-20 inoculant; JF: MCMB application; JS: biochar-based agent application.

In this study, 61,834,420–81,577,396 raw reads and 60,425,910–79,749,810 clean reads were generated from 12 soil samples. The rarefaction curves gradually plateaued (Figure 2a), indicating an adequate depth for capturing most of the taxa.

Different treatments exerted significant effects on the α diversity of soil microbial communities (Table 5). The Chao1 richness exhibited the following order: SS > JS > JF > CK. Specifically, SS demonstrated a significant enhancement over CK and JF by 4.73% and 3.24%, respectively (*p* < 0.05), while JS also exceeded CK by 3.26% (*p* < 0.05). In terms of Shannon diversity, SS achieved the highest value (6.92), significantly surpassing those of CK and JS (*p* < 0.05). However, no significant differences were observed in Simpson diversity or Pielou’s evenness across the treatments. A two-way ANOVA indicated that bacteria presence significantly increased Chao1 richness (*p* < 0.001), along with a significant Biochar × Bacteria interaction (*p* = 0.038). No significant interactions were found for Shannon and Simpson diversity or Pielou’s evenness (*p* > 0.05), and thus, simple-effect analyses were not conducted for these indices.

PLS-DA revealed that the treatments significantly shaped the microbial community structure, as confirmed by PERMANOVA (F = 2.03, R^2^ = 0.43, *p* = 0.023; Figure 2b). PC1 and PC2 explained 17.0% and 9.7% of the total variance in the microbial community structure, respectively. Samples from CK, JF, and JS were distributed along the positive PC1 axis, whereas the SS samples clustered along the negative PC1 axis and were clearly separated from the other treatments. JS and CK samples were positioned on the positive and negative sides of the PC2 axis, respectively. NMDS analysis showed similar trend (Figure S2a). Furthermore, dissimilarity analysis at the species level confirmed that the community structures of the JF and JS treatments differed significantly from that of CK (*p* < 0.001 and *p* < 0.05, respectively), and a significant difference was detected between the SS and JF treatments (*p* < 0.01, Figure 2c).

#### 2.5.2. Microbial Community Composition

**Figure 3:**
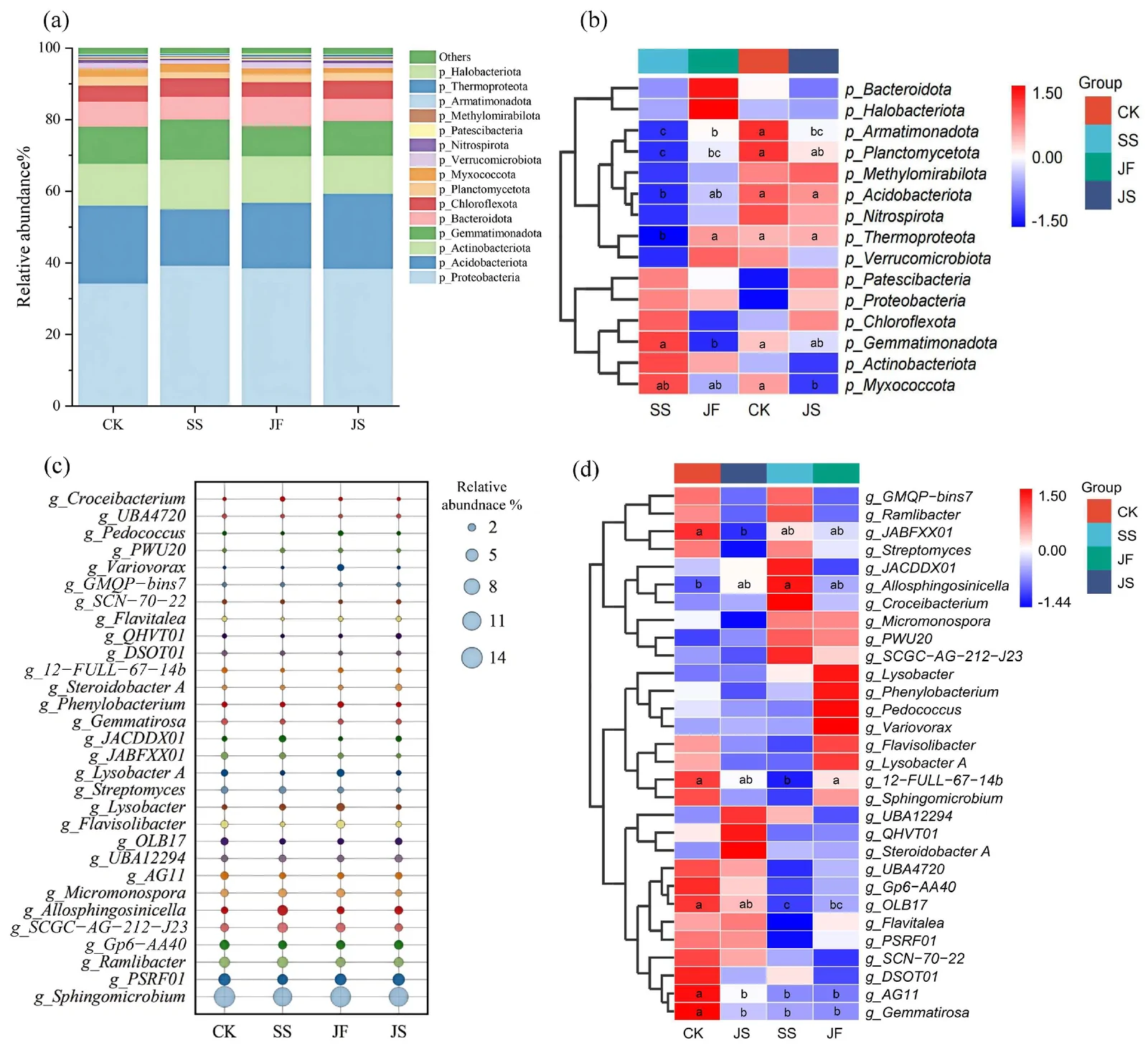
Histogram (**a**) and heatmap (**b**) of the top 15 dominant phylum-level taxa; bubble plot (**c**) and heatmap (**d**) of the top 30 dominant genus-level taxa. Statistical comparisons were conducted via one-way ANOVA (LSD) or Welch’s ANOVA (Games–Howell) according to Levene’s variance homogeneity test. Different lowercase letters indicate significant differences among treatments (LSD test, *p* < 0.05). CK, blank control; SS, application of SS-20 inoculant; JF, MCMB application; JS, application of biochar-based agent.

Metagenomic profiling revealed 215 phyla, 531 classes, 1574 orders, 3587 families, 12,008 genera, and 32,006 species across all the soil samples. The amendments exerted distinct effects on the soil microbial community composition. The dominant phyla (>5% relative abundance) included *Proteobacteria* (34.16–39.18%), *Acidobacteriota* (18.25–21.82%), *Actinobacteriota* (10.65–13.85%), *Gemmatimonadota* (8.39–11.22%), and *Bacteroidota* (6.21–8.19%), with *Proteobacteria* being the most abundant (Figure 3a). Although the amendments did not substantially alter the overall phylum-level composition, they significantly altered the community diversity patterns (Figure 3b). Specifically, the relative abundances of *Armatimonadota*, *Planctomycetota*, *Acidobacteriota*, and *Thermoproteota* decreased, whereas the relative abundances of *Gemmatimonadota* (11.22%) and *Myxococcota* (2.32%) peaked in the SS among all treatments. *Acidobacteriota* and *Thermoproteota* tended to be enriched in the JS group.

At the genus level, the amendments similarly altered the relative abundance and diversity of the microbial communities (Figure 3c,d). The core dominant genera (>2%) included *Sphingomicrobium* (10.61–13.17%), *PSRF01* (*Acidobacteriota*; 3.34–4.48%), *Ramlibacter* (2.79–3.49%), and *Gp6-AA40* (*Acidobacteriota*; 2.25–2.43%). The SS group exhibited a high abundance of *Allosphingosinicella* (3.17%) but low abundances of the *Acidobacteriota*-affiliated taxa *12-FULL-67-4b* (0.61%) and *OLB17* (1.17%). Compared with those in CK, the relative abundances of *AG11* (*Gemmatimonadota*) and *Gemmatirosa* were significantly lower in the SS, JF, and JS treatments (*p* < 0.05), and the abundance of *JABFXX01* (belonging to *Myxococcota*) in JS was 44.35% lower (*p* < 0.05).

### 2.6. Correlations Between Environmental Factors and Microbial Communities

Pearson correlation analysis was performed between the relative abundances of dominant taxa and soil factors at both the phylum and the genus level (Figure S1). No correlations remained significant after FDR correction at either level. At the uncorrected level, however, consistent trends emerged: soil pH was positively correlated with *Planctomycetota* at the phylum level and with *AG11*, *Gp6_AA40* and *OLB17* at the genus level, whereas TP content was negatively correlated with *Verrucomicrobiota*, *OLB17*, and *Lysobacter A*, respectively. Additional associations included *Gemmatimonadota* with EC, *Ramlibacter* with salt content, and *Allosphingosinicella* with S-CAT activity.

**Figure 4:**
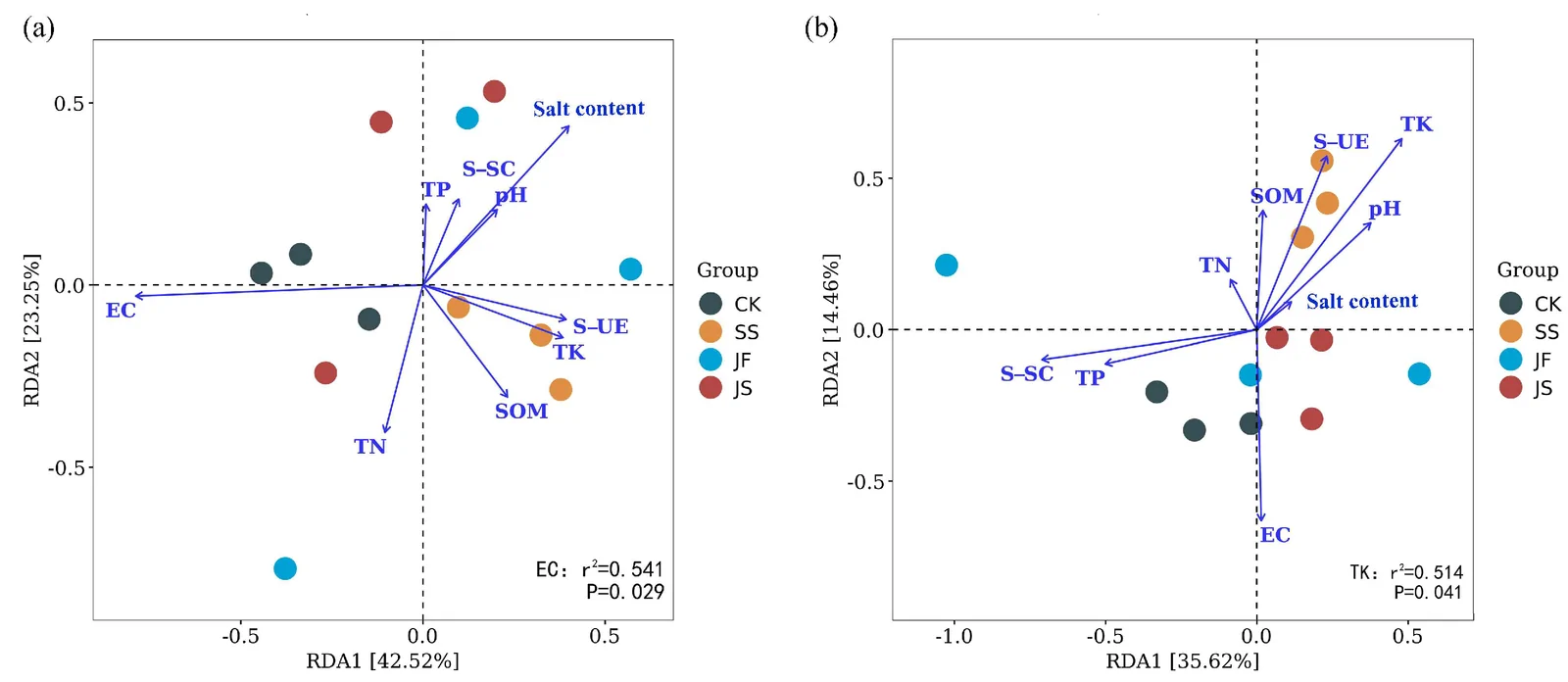
RDA analysis of microbial community and environmental factors at phylum level (**a**) and genus level (**b**).

Furthermore, VIF analysis was applied to remove multicollinear environmental factors. At the phylum level (Figure 4a), RDA1 and RDA2 explained 42.52% and 23.25% of the total variance, respectively (approximately 65.77%). TN content and EC were negatively correlated with RDA1, whereas all the other factors except TP were positively correlated with RDA1. S-SC, salt content, pH, and TP were positively associated with RDA2, while S-UE, TK, TN, and SOM were negatively correlated. EC was identified as the primary factor significantly structuring the phylum-level community (*p* < 0.05). At the genus level (Figure 4b), RDA1 and RDA2 explained 35.62% and 14.46% of the variance, respectively (approximately 50.08%). TP, S-SC, and TN were negatively correlated with RDA1, whereas all the other factors except EC and SOM were positively associated with RDA1. TP, S-SC, and EC were negatively correlated with RDA2, whereas the remaining factors were positively correlated. TK content served as the significant driving factor for the genus-level community structure (*p* < 0.05).

### 2.7. Analysis of Functional Potentials of Microbial Communities

**Figure 5:**
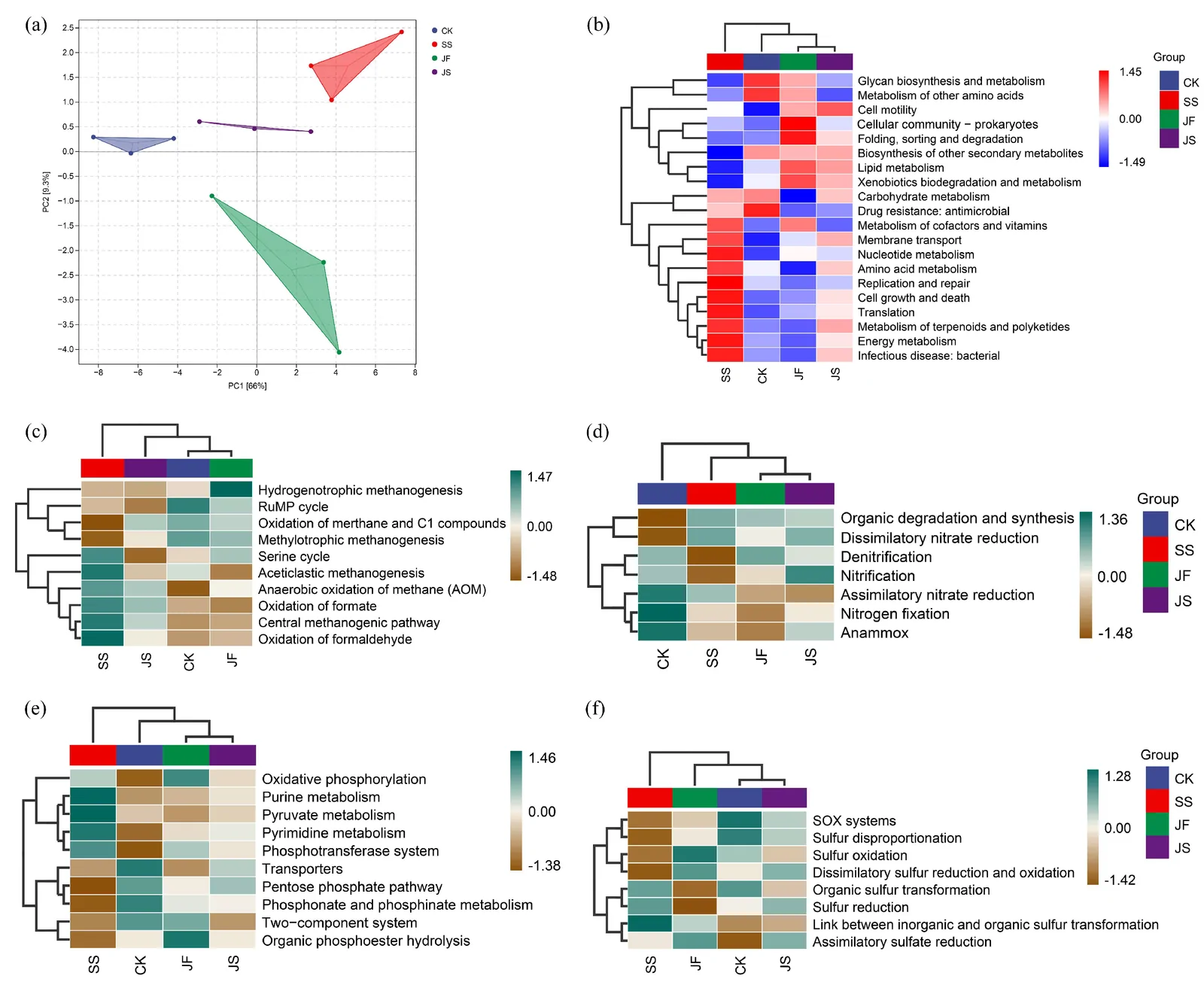
PLS-DA at KEGG L2 level (F = 5.159, R^2^ = 0.659, *p* = 0.011), (**a**); Effects of different treatments on genes related to KEGG L2 pathways (**b**), cycles of carbon (**c**), nitrogen (**d**), phosphorus (**e**), and sulfur (**f**). CK: blank control; SS: SS-20 inoculant application; JF: acid modified biochar application; JS: biochar-based agent application.

Different treatments significantly affected the functional potential of soil microbial communities at the KEGG L2 level (F = 5.159, R^2^ = 0.659, *p* = 0.011; Figure 5a). PC1 and PC2 explained 66.0% and 9.3% of the total variance in functional profiles, respectively. The CK samples were predominantly located on the negative side of PC1, whereas the SS samples were positioned on the positive side. NMDS analysis corroborated these findings, demonstrating that the treatments significantly altered the functional structure at the KEGG L2 level (Figure S2b).

The relative abundance of genes assigned to KEGG L2 categories differed across treatments (Figure 5b). Specifically, genes related to nucleotide metabolism, replication and repair, translation, and cell growth and death were enriched in the SS group. Genes associated with antimicrobial drug resistance, metabolism of other amino acids, and glycan biosynthesis and metabolism were more prevalent in CK. Genes linked to cellular community–prokaryotes and those involved in folding, sorting, and degradation were enriched in the JF group, while genes related to cell motility exhibited the highest relative abundance in the JS group.

Carbon, nitrogen, phosphorus, and sulfur cycling are key biogeochemical processes that sustain soil fertility and ecosystem stability. In the carbon cycle (Figure 5c), genes related to formaldehyde oxidation, the central methanogenic pathway, and aceticlastic methanogenesis tended to be enriched in SS, whereas genes related to hydrogenotrophic methanogenesis increased in JF. No marked enrichment was evident in CK or JS groups. In the nitrogen cycle (Figure 5d), genes related to assimilatory nitrate reduction, nitrogen fixation, and anammox were abundant in CK, and nitrification-related genes were enriched in JS. With respect to phosphorus cycle (Figure 5e), genes associated with purine and pyruvate metabolism showed an enrichment pattern in SS, organic phosphoester hydrolysis genes tended to be enriched in JF, and no clear enrichment was detected in JS. In the sulfur cycle (Figure 5f), genes linking inorganic and organic sulfur transformation were abundant in SS, sulfur oxidation genes were enriched in JF, and the genes of SOX system dominated in CK.

**Figure 6:**
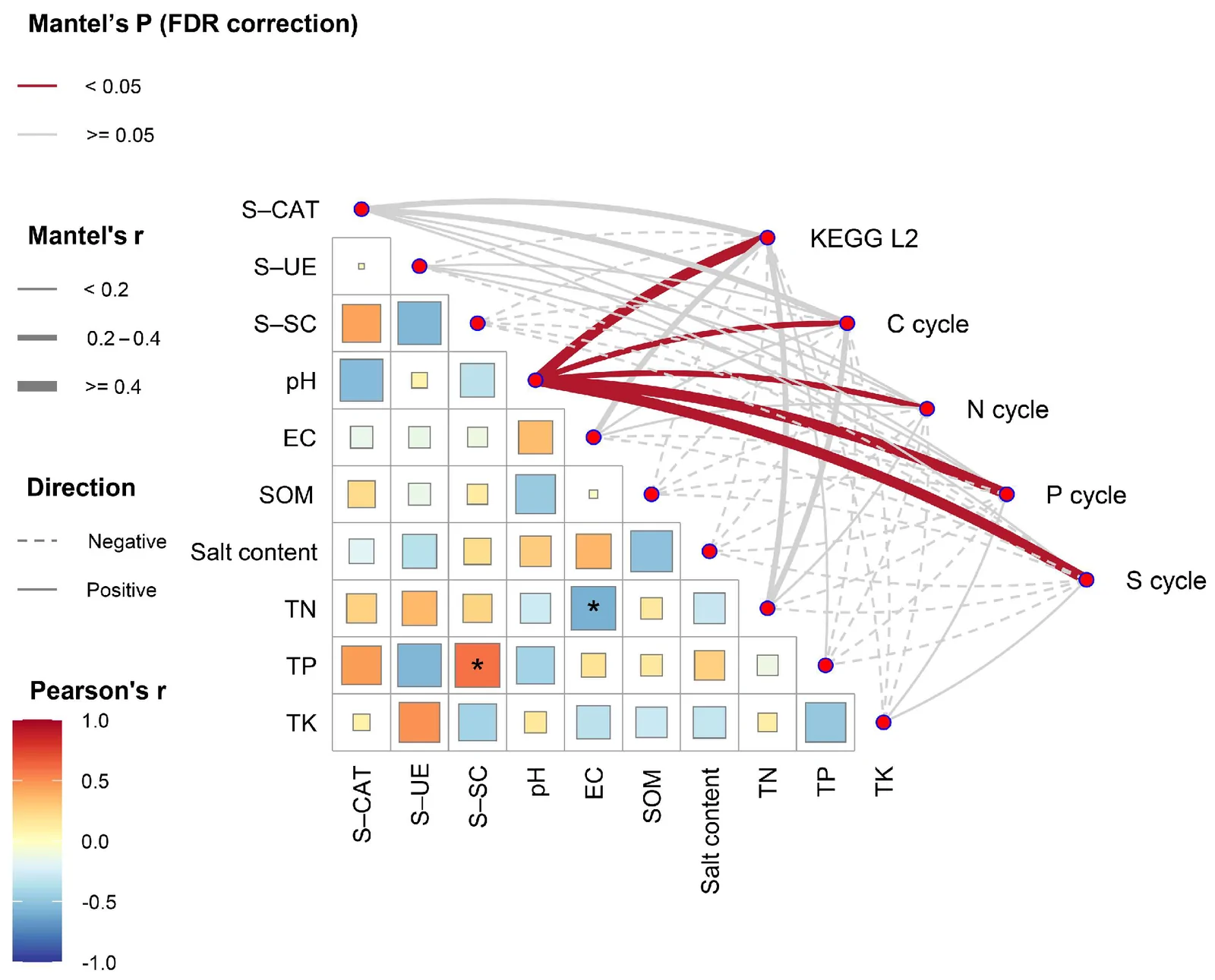
Mantel test analysis between soil factors and functional gene relative abundances (KEGG L2 and C, N, P, and S cycles at pathway level). Lower-left triangle: Pearson correlations among soil factors; box color indicates correlation direction. Right: functional gene nodes. Lines connecting soil factors and functional genes represent Mantel correlations computed using Pearson’s method; line color indicates Benjamini–Hochberg FDR-corrected adjusted *p* values (red, *p* < 0.05; grey, *p* ≥ 0.05), line thickness indicates Mantel’s r, and line style indicates correlation direction (solid, positive; dashed, negative). *: Based on uncorrected *p* values; no correlations remained significant after adjustment (all adjusted *p* > 0.05).

Furthermore, Mantel test (Figure 6) revealed that soil pH was the only factor significantly correlated with all five functional gene communities after FDR correction (adjusted *p* < 0.05). None of the other soil factors reached significance (adjusted *p* > 0.05). Pearson correlation analysis revealed generally moderate associations among soil factors, with the soil pH covarying to some extent with SOM and TP content (all adjusted *p* > 0.05).

## 3. Discussion

### 3.1. Characteristics of the Biochar-Based Agent

In this study, citric acid dissolves soluble cations in this ash [21,22], unclogging micropores and altering the mineral composition of MCMB [23]. Cao et al. [24] reported a similar outcome in which citric acid dehydrogenation of ash in the biogas residue biochar resulted in unclogged micropores and increased the specific surface area from 96.60 to 266.2 m^2^/g. The ready acidification of the 400 °C biochar indicates that its alkalinity is derived mainly from weakly basic salts or surface-adsorbed alkali neutralized by protons [25], rendering it a suitable immobilization carrier. Acid modification also altered the hydroxyl structures and mineral phases of MCMB. The mineral-phase changes presumably arise from FeSO_4_ addition, as chicken manure biochar is rich in inorganic minerals and protein-derived nitrogen groups that coordinate Fe^2+^ [26], and the introduced Fe^2+^ may promote the formation of hydroxyl coordination structures and mineral phases [27]. Free –OH groups form intermolecular hydrogen bonds with polar groups on microbial membranes, favoring rapid and stable colonization [28]. In the future study, XRD or XPS results may facilitate to confirm the chemical changes caused by citric acid–FeSO_4_ modification. Because pore structure, specific surface area, and functional groups govern microbial loading capacity [29], the immobilization ratio should not be too high or too low: 5% biochar resulted in the highest per-gram loading but left surplus cells in suspension, whereas 15% biochar resulted in wasted material through redundant adsorption sites; thus, an optimal biochar-to-inoculant ratio exists (10% in this study, Table S3).

### 3.2. Effects of Biochar-Based Agent on Vegetable Growth and Soil Properties

Exogenous amendments generally improved pakchoi growth, and JS performed best. The plant height and aboveground and underground biomass were greatest under JS treatment, differing significantly from CK, SS, and JF (Table 1). Biochar can moderately increase soil N, P, and K levels and carries mineral nutrients that sustain root energy metabolism and dehydrogenase activity [30]; its porous structure offers attachment sites and niches for both the inoculated SS-20 strain and indigenous microbes, improving the rhizosphere microenvironment. These findings coincide with the data on root activity, which remained higher in JF and JS than in CK and SS and peaked in the JS (0.572 μg/min/g). The MDA and Pro contents and SOD and POD activities did not significantly differ among treatments, although JS trended toward having the lowest MDA content and the highest Pro content and SOD and POD activity. In the present study, the antioxidant system may already have been fully induced under the imposed stress, leaving little room for further elevation; biochar-mediated adsorption may buffer transient ion shocks without being strong enough to alter membrane peroxidation [31]; a single sampling time may also miss an enzyme response that shifts with developmental stage and time. The present data cannot distinguish among these possibilities, which requires larger samples, additional stress indicators and time-course sampling. JS therefore alleviated salinity–alkalinity stress mainly by improving the soil environment and promoting root development [32], whereas any contribution from enhanced antioxidant defense remains to be established.

S-CAT, a sensitive indicator of microbial metabolic intensity, was significantly higher in both SS and JS than in CK and JF. SS-20 may release signaling compounds, including organic acids and amino acids, during metabolism [33], stimulating microbial proliferation and activity, while biochar supplies carbon substrates and colonization niches for enzyme-producing microorganisms. Their combined action likely explains the increase in S-CAT content in SS and JS, and the S-CAT content was marginally stronger in JS. JS treatment resulted in the highest SQI (0.614, a 96.23% increase over that of CK). Shapley analysis attributed 55.67% of this increase to MCMB and 44.33% to SS-20, with a synergy value of only +0.004. The amendments thus acted in a nearly additive manner, with biochar as the main driver of soil improvement and SS-20 as a supplementary component, a pattern echoed by Hou et al. [34], in which biochar acted as the foundational component and the microbial agent depended on the environment biochar created. This near-additive behavior at the soil level does not preclude interactive effects on the plant: a significant biochar × bacteria interaction was detected for aboveground fresh weight (*p* = 0.045), soil pH (*p* = 0.000), EC (*p* = 0.010), and TP content (*p* = 0.050).

### 3.3. Composition and Structure of Soil Microbial Community

The relative abundance of *Proteobacteria* tended to decrease in the order of SS > JF > JS > CK and tended to be negatively correlated with soil pH (Figure 3 and Figure S1). Most *Proteobacteria* favor near-neutral conditions; the amendment-associated pH reduction may have alleviated alkaline stress and contributed to their increased abundance. High S-SC activity generally reflects a stable soil environment with active carbon metabolism [35]. Accordingly, the relative abundance of *Bacteroidota*, a typical oligotrophic taxon adapted to nutrient limitation, was lowest in the JS group, where S-SC activity was increased. *Myxococcota* was associated with high-EC, high-salinity soils, possibly because its cell membranes are rich in ether lipids and cyclopropane fatty acids, which help maintain membrane fluidity under osmotic stress from up to 10 g/kg Na^+^ and Cl^−^ [32]. The lowest *Myxococcota* abundance in the JS treatment (Figure 3b) may thus reflect substantial salinity reduction, which created conditions less conducive to salt-tolerant populations.

At the genus level, *Allosphingosinicella* may harbor inorganic P-solubilizing genes (e.g., *gcd* and *pqqB*) and a phosphatase gene (e.g., *phoN*) [36]. In high-TP soils, e.g., under SS treatment, these bacteria may mobilize insoluble P by secreting gluconic acid and phosphatases, potentially increasing the available P and supporting their own proliferation [36]. High S-SC activity, the SOM and TP levels indicate eutrophic conditions, which may account for their negative associations with oligotrophic genera such as *Flavisolibacter*, *Lysobacter A*, and *Streptomyces*. The denitrifying genus *UBA12294* [37] (belonging to *Chloroflexota*) has been reported to strengthen connections with anammox bacteria and facilitate biofilm formation [38]; whether its enrichment in JS contributes to N cycling requires further analysis.

RDA revealed that soil EC and TK content were the key drivers shaping microbial community structure at phylum and genus levels, respectively. As a comprehensive index for soil salinity, EC regulates microbial communities via osmotic stress and ion toxicity. Abdalla et al. [39] also emphasized EC as the primary factor driving microbial community divergence in saline–alkali soils. As an essential macronutrient for microorganisms, K structures microbial communities by modulating enzyme activity, maintaining osmotic homeostasis, and shaping nutritional niches. The TK content was the highest in the JS group (1.93%, Table 3). This finding aligns with previous studies from Sun et al. [4], highlighting the key role of nutrient availability in structuring microbial communities. However, studies have reported that TN, TP, and saline–alkali stress indicators (e.g., pH, EC, and ESP) are the more critical restrictive driving factors [40,41]. These contrasting findings suggest that the mechanisms governing the interactions between soil TK content and microbial communities are complex and merit further investigation.

### 3.4. Functional Potential of Soil Microbial Community

Different treatments significantly altered the functional potential of the soil microbial community at the KEGG L2 level. High abundances of genes involved in nucleotide metabolism, replication and repair, and translation reflect active proliferation and protein synthesis, or DNA repair under stress [13]; direct SS-20 inoculation may have triggered short-term competition and defense responses in the indigenous community. High abundances of genes involved in cell motility reflect the microbial capacity for migration [42], and MCMB micropores in JS may have offered niches for highly motile microorganisms to gain a competitive advantage [43].

With respect to carbon cycling, the inoculated *Bacillus* strain likely harbors formate dehydrogenase genes [3], potentially explaining the increase in functional potential of formate oxidation in SS. Central and aceticlastic methanogenesis are typically mediated by anaerobic methanogens [44]; whether their enrichment is linked to SS-20 metabolism requires verification. Genes for hydrogenotrophic methanogenesis (H_2_-dependent CO_2_ reduction to CH_4_ [44]) were enriched in JF, presumably reflecting MCMB-provided microhabitats and substrates.

With respect to nitrogen cycling, the low amount of available N in CK may have driven the microbial conversion of nitrate to organic N via assimilatory reduction, an N-conservation strategy [45], while N-fixing and anammox bacteria were more competitive under N-deficient, anaerobic conditions, explaining their gene enrichment in CK. Nitrification, a key soil N-cycle process [46], genes of which were enriched in JS, probably owing to the high soil N content (0.18%) supplying ample substrate for nitrifiers [47].

With respect to phosphorus cycling, enriched purine and pyrimidine metabolism genes indicate active DNA replication and RNA transcription, which is consistent with the L2 results. Phosphoesterase secretion hydrolyzes organic P into bioavailable inorganic P [48], and MCMB may create favorable microhabitats for phosphoesterase producers [49], promoting organic P mineralization in the JF group.

With respect to sulfur cycling, elevated genes linked to inorganic and organic S transformations in SS suggest increased sulfur bioavailability and turnover. In the JS group, MCMB-immobilized SS-20 may occupy niches and suppress indigenous sulfate-reducing bacteria. The SOX system oxidizes sulfur substrates directly without intermediate accumulation, sustaining S cycling in carbon-poor environments [50]; without exogenous input, chemolithoautotrophic sulfur oxidizers in CK likely gained a survival advantage via this system.

The Mantel test result supports the established view that soil pH is a key driver of microbial communities. Even subtle pH gradients caused significant divergence in genes related to C, N, P, and S cycles, indicating the high pH sensitivity of functional microbes. Consistent with the findings of Hagh-Doust et al. [51], compared with anthropogenic disturbances such as N deposition, pH more strongly shaped the C, N, and P cycling gene composition. However, the small sample size (*n* = 12) limited the power of the Mantel test and may have masked nonlinear effects; future work should expand sampling and use structural equation modeling or random forests to disentangle direct and indirect effects.

In the present study, pokchoi growth, soil properties, microbial communities, and nutrient cycling potential during saline–alkaline soil remediation were characterized. Several limitations should be acknowledged. First, the differential distribution of element-cycling genes across treatments provides mechanistic hypotheses for amendment-mediated nutrient dynamics, which require validation via direct analyses such as soil respiration, metabonomics, and meta-transcriptomic analyses. Second, direct evidence of SS-20 colonization is lacking. The activity and colonization of the SS-20 strain in bulk soil and rhizosphere soil should be monitored using technologies such as qPCR, fluorescence in situ hybridization, and meta-transcriptomics. Nevertheless, the pot experiments simulated only topsoil effects and did not fully represent field conditions, where deeper soil layers are involved. In the field, amendment-derived nutrients and microorganisms may migrate downward while salt ions from deeper layers rise into the plow layer; field trials are thus needed to determine the optimal amendment dosage and application scope under realistic saline–alkaline conditions.

## 4. Materials and Methods

### 4.1. Preparation of Biochar-Based Microbial Agent

#### 4.1.1. Preparation of MCMB

Based on preliminary screening of straw, cow dung, pruning residue, and chicken manure biochars, chicken manure biochar (400 °C) was selected as the carrier matrix because its pH approximately neutrality (7.08) after acid modification. The biochar was produced via slow pyrolysis in a programmable tube furnace (Jinan Jingrui Instrument Co., Ltd., Jinan, China). Briefly, air-dried chicken manure was pyrolyzed at 400 °C for 3 h under an N_2_ atmosphere at a heating rate of 10 °C/min.

The MCMB preparation was performed as follows: firstly, citric acid was slowly added to deionized water at a ratio of 1:50 (*w*/*v*) to achieve a citric acid solution. FeSO_4_ was then slowly added to citric acid solution at a ratio of 1:20 (*w*/*v*), followed by 30 min stirring at 25 °C. Raw chicken manure biochar was immersed in the above solution at a ratio of 1:20 (*w*/*v*), followed by 10 h stirring at 25 °C. The impregnated biochar was washed repeatedly with deionized water until the filtrate pH constant, oven-dried at 80 °C for 12 h, and then placed in a tube furnace for secondary pyrolysis. The furnace was heated gradually at a rate of 10 °C/min to 400 °C under N_2_ flow and maintained for 1 h. After cooling naturally to ambient temperature, the MCMB was obtained. The physicochemical properties of biochar before and after modification were determined (Supplementary Material).

#### 4.1.2. Preparation of Biochar-Based SS-20 Agent

The SS-20 suspension was adjusted to an optical density of 1.0 at 600 nm and mixed with MCMB at a ratio of 10% (*w*/*v*) (Table S3). The mixture was incubated at 30 °C with shaking at 120 r/min for 12 h. Immobilization efficiency was determined by the plate dilution method. The loading rate (*R*, %) and bacterial load per gram of MCMB (*LPG*, CFU/g) were calculated as:

(1) $$R=\frac{C0V0-C1V1}{C0V0}\times 100\%$$

(2) $$\mathrm{LPG}=\frac{C0V0-C1V1}{W}$$

where *C*0 and *C*1 are the bacterial concentrations (CFU/mL) before and after immobilization, *V*0 and *V*1 are the corresponding volumes (mL), and *W* is the dry weight of MCMB (g). The microstructure of the agent was characterized by cryo-scanning electron microscopy (Cryo-SEM, FEI Quanta 450, Thermo Fisher Scientific/FEI, Hillsboro, OR, USA).

### 4.2. Experimental Design

The experimental soil was classified as sandy loam, collected from the plow layer (0–20 cm depth) of a field cultivated with *Brassica chinensis* L. for 5 consecutive years in Zhuanghang Town, Fengxian District, Shanghai (30°53′ N, 121°23′ E). The main physicochemical properties are summarized in Table 6.

The pakchoi cultivar tested was Xinaiqing (*Brassica chinensis* L.). A pot experiment was conducted from 20 May to 30 June 2025 with four treatments: (i) blank control (CK); (ii) inoculation with *Bacillus velezensis* SS-20 suspension (SS); (iii) application of microbial compound microbial biochar (MCMB) (JF); and (iv) application of the biochar-based microbial agent (JS). Each plastic pot (25 cm diameter × 20 cm height) was filled with 3.0 kg of experimental soil. For the SS group, the SS-20 strain was cultivated, harvested, and resuspended in sterile distilled water to obtain a concentrated cell suspension of 3.0 × 10^11^ CFU/mL. A 40 mL suspension was applied to each pot, delivering approximately 1.2 × 10^13^ CFU per pot. In JS, the biochar was loaded with SS-20 cells according to Section 4.1.2 (the original cell density of approximately 6.3 × 10^10^ CFU/mL), achieving a concentration of 1.16 × 10^13^ CFU per pot. Biochar was thoroughly mixed with soil at a mass ratio of 1:100 in the JF and JS treatments. The CK, JF, and JS pots received an equal volume of sterile water. Compound fertilizer (N–P_2_O_5_–K_2_O, 15-15-15) was applied as inorganic fertilizer at 3.0 g per pot and thoroughly mixed with the soil before planting. Pakchoi seedlings of uniform vigor were transplanted into the pots, with six seedlings retained per pot. All treatments were arranged in a randomized design with three replicates in a greenhouse (25–30 °C, natural light, 40–50% relative humidity) and rotated weekly to minimize positional effects. No pesticides or herbicides were applied during the experiments.

The plant and bulk soil samples were harvested after 45 days. Five soil samples of 0–20 cm depth from each pot were collected, and fully homogenized into a single one. One portion was frozen and stored at −80 °C for high-throughput microbial sequencing, and the remaining soil samples were air-dried, ground, and sieved for subsequent determination. Plant samples from each pot were collected separately and washed to remove surface contamination; the agronomic traits and stress resistance indicators were measured in this study.

### 4.3. Sample Analysis

#### 4.3.1. Analysis of Vegetable Indexes

Plant height was measured using a ruler. The fresh weight was weighed immediately after harvesting using an electronic balance. Roots were thoroughly rinsed with deionized water, surface-dried, and weighed to determine root fresh weight. For dry weight determination, samples were first deactivated at 105 °C for 30 min and subsequently oven-dried at 85 °C to a constant weight, after which dry biomass was recorded.

The chlorophyll content was estimated non-destructively in situ using a portable chlorophyll meter (SPAD-502, Konica Minolta, Tokyo, Japan). Root activity was determined as root dehydrogenase activity using the 2,3,5-triphenyltetrazolium chloride method with a commercial assay kit (Comin Biotechnology Co., Ltd., Suzhou, China).

Regarding plant stress resistance, the activities of SOD and POD, as well as the contents of MDA and Pro, were determined by nitroblue tetrazolium photoreduction method, guaiacol method, thiobarbituric acid reaction, and acid ninhydrin method, respectively, using commercial assay kits (Comin Biotechnology Co., Ltd., Suzhou, China). For each parameter, three representative plants were randomly selected from each pot and measured; the mean value was calculated as the replicate value. Statistical analysis was performed with *n* = 3 at the plot level.

#### 4.3.2. Analysis of Soil Properties and Enzyme Activities

Soil pH and EC were measured in a 1:2.5 (*w*/*v*) soil-to-water suspension using a pH/conductivity meter (SevenCompact, Mettler Toledo, Greifensee, Switzerland) [18]. Soil TP and TK were determined after digestion with HNO_3_, HClO_4_, and HF by inductively coupled plasma atomic emission spectrometry (ICP-AES; PlasmaQuant 9100, Jena, Germany). Soil salt content was determined by the gravimetric (residue-on-ignition) method in accordance with LY/T 1251-1999 [52]. SOM content was quantified by the potassium dichromate oxidation–external heating method, and TN was determined by the Kjeldahl digestion method [53].

Soil enzyme activities were measured using microplate-based colorimetric assays with a microplate reader (Infinite 200 Pro, Tecan, Männedorf, Switzerland). The activities of soil sucrase (S-SC) and soil urease (S-UE) were determined by the 3,5-dinitrosalicylic acid colorimetric method and indophenol blue colorimetric method, respectively. Soil catalase (S-CAT) activity was determined by measuring absorption attenuation of H_2_O_2_ at 240 nm wavelength. All measurements were performed in three independent replicates.

#### 4.3.3. Evaluation of the Soil Quality Index and Contribution Rate

A total of 10 soil indicators (soil physicochemical properties and enzyme activities) were included in the SQI calculation. PCA was performed on the standardized indicator matrix [17,18] and is presented in the Supplementary Material. Each selected indicator was converted to a dimensionless score (0–1) using Min-Max normalization, with the scoring direction determined by the biological relationship with soil quality (Supplementary Material). According to PCA [54,55], the weight is equal to the ratio of the common factor variance of each indicator to the sum of the common factor variance of all indicators. The SQI was calculated for each treatment group according to Equation (3):

(3) $$\mathrm{SQI}=\sum_{i=1}^{n}\mathrm{WiSi}$$

where *SQI* is the soil quality index; *n* is the number of soil indicators; *Wi* is the indicator weight value (Table S5); *Si* is the linear or nonlinear score of the *i* indicator. Higher SQI indicates higher soil quality. The SQI improvement rate (*IR*) of each treatment relative to CK was then calculated as:

(4) $$\mathrm{IRi}=\frac{\bar{\mathrm{SQI}}i-\bar{\mathrm{SQI}}\mathrm{ck}}{\bar{\mathrm{SQI}}\mathrm{ck}}\times 100\%$$

where $\bar{\mathrm{SQI}}i$ indicates the average *SQI* in *i* treatment; $\bar{\mathrm{SQI}}\mathrm{ck}$ is the average *SQI* in CK treatment. In addition, Shapley values (φ) were calculated (Equations (5) and (6)), in order to decompose the relative contributions of the two amendment factors, e.g., SS-20 inoculant and biochar, to the *SQI* improvement in the JS relative to CK. For the two-factor case:

(5) $$\varphi A=1/2[\left(\bar{\mathrm{SQI}}\mathrm{ss}-\bar{\mathrm{SQI}}\mathrm{ck}\right)+(\bar{\mathrm{SQI}}\mathrm{js}-\bar{\mathrm{SQI}}\mathrm{jf})]$$

(6) $$\varphi B=1/2[\left(\bar{\mathrm{SQI}}\mathrm{jf}-\bar{\mathrm{SQI}}\mathrm{ck}\right)+(\bar{\mathrm{SQI}}\mathrm{js}-\bar{\mathrm{SQI}}\mathrm{ss})]$$

The contribution rate of each factor was calculated as $\frac{\varphi i}{\varphi A+\varphi B}\times 100\%$. This approach provides an unbiased attribution of the combined treatment effect to individual factors [56].

#### 4.3.4. Soil DNA Extraction and Metagenome Sequencing

Total microbial genomic DNA samples were extracted using the MagBeads FastDNA Kit for Soil (116564384) (MP Biomedicals, Salon, OH, USA), following the manufacturer’s instructions. DNA quantity and quality were measured using a Qubit™ 4 Fluorometer (Q33231, Invitrogen, Carlsbad, CA, USA) and agarose gel electrophoresis, respectively. Then, DNA was processed to construct metagenome sequencing libraries with insert sizes of ~400 bp. It was sequenced by Illumina NovaSeq platform (Illumina, San Diego, CA, USA) with PE150 strategy at Personal Biotechnology Co., Ltd. (Shanghai, China).

Raw reads were processed to obtain quality-filtered reads for further analysis. After adapter removal and trimming of low-quality reads using fastp (v 0.23.2), MEGAHIT (version 1.1.2) was used to concatenate and assemble the valid reads sequences [57]. Prodigal (version 2.6.) [58] was used to identify open reading frames and predict the coding regions. Taxonomic classification was executed using Kraken2 (version 2.0.8-beta) [59] against the customized databases. Using mmseq2 (v 13.45111), functional gene annotations were performed on databases such as KEGG, MCyc, NCyc, PCyc, and SCyc to obtain species abundance tables at various classification levels and abundance tables at various functional levels. The raw sequence data have been deposited in the Genome Sequence Archive (GSA: CRA049546) that are publicly accessible at https://ngdc.cncb.ac.cn/gsa (accessed date 26 September 2026).

### 4.4. Statistical Analysis

All the statistical analyses were performed using SPSS 26.0 (IBM, Armonk, NY, USA), R 4.2.1 (Vienna, Austria), and Origin 2023 (OriginLab, Northampton, MA, USA). Homogeneity of variance was tested using Levene’s test. One-way ANOVA followed by LSD test was used for pairwise comparisons among the four treatments. For variables that did not meet homogeneous assumptions, Welch’s ANOVA followed by the Games–Howell post hoc test was applied. For the vegetable indices, soil physicochemical properties, enzyme activities, and microbial α diversity indices, two-way ANOVA was performed to test the main effects of the inoculant and biochar, as well as their interaction effects. When the interaction was significant (*p* < 0.05), simple effect analysis was conducted to examine the effect of one factor at each level of the other factor.

Taxonomic β-diversity of the microbial community was evaluated using Partial Least Squares Discriminant Analysis (PLS-DA) and Non-Metric Multidimensional Scaling (NMDS) at the order level, with further analysis conducted through distance-difference analysis at the species level. Functional β-diversity was similarly assessed using PLS-DA and NMDS, based on KEGG L2 functional profiles. To determine the significance of community differences among treatments, Permutational Multivariate Analysis of Variance (PERMANOVA) was employed with 999 permutations. Pearson correlation analysis was utilized to assess the relationship between dominant microbial taxa and soil properties, considering correlations with adjusted *p*-values < 0.05 as significant, following the Benjamini–Hochberg False Discovery Rate (FDR) correction method. Environmental factors exhibiting a variance inflation factor (VIF) greater than 10 were excluded to mitigate multicollinearity. Subsequently, the relationships between soil environmental factors and microbial community structure were investigated using Redundancy Analysis (RDA) with the “vegan” package in R. Differential analysis of microbial community functional potential was performed based on functional annotation databases, with results visualized through heatmaps. Meanwhile, Mantel test was employed to examine the correlations between soil factors and microbial functional genes. The soil factors were standardized using the Z-score, while the dissimilarity between microbial communities was assessed via the Bray–Curtis algorithm. A correlation analysis was performed utilizing the Pearson method, with adjustments made for multiple comparisons using the FDR correction.

## 5. Conclusions

In this study, a biochar-based *Bacillus velezensis* SS-20 agent was developed, demonstrating ameliorative effects on both pakchoi production and the properties of saline–alkali soils. The JS treatment notably enhanced the height and biomass yield of pakchoi plants and showed a tendency to improve plant stress resistance. Regarding soil characteristics, JS treatment resulted in a reduction in soil pH, EC, and salt content, while significantly increasing soil catalase activity. Metagenomic analysis further indicated that JS treatment influenced the soil microbial community and its functional potential, with EC and TK identified as the primary determinants of community structure at the phylum and genus levels, respectively. These findings suggest that the biochar-based *B*. *velezensis* agent enhances pakchoi yield and quality through an additive interaction by improving soil environmental conditions, which subsequently modulate the abundance of dominant microbial taxa and their functional potential. Long-term field trials are necessary to determine the optimal dosage and application parameters of JS under practical saline–alkali conditions.

## Figures and Tables

**Table 1:** Analysis of agronomic traits of *Brassica chinensis* L.

Treatment	Fresh Weight (g)		Dry Weight (g)		Plant Height (cm)
	Aboveground	Underground	Aboveground	Underground	
CK	4.79 ± 1.33 b	0.27 ± 0.07 b	0.43 ± 0.27 b	0.021 ± 0.006 b	11.05 ± 1.34 b
SS	5.58 ± 0.75 b	0.33 ± 0.03 b	1.06 ± 0.228 b	0.035 ± 0.016 b	12.30 ± 0.92 ab
JF	5.15 ± 0.88 b	0.36 ± 0.06 b	0.91 ± 0.40 b	0.034 ± 0.008 b	11.59 ± 0.58 b
JS	8.46 ± 0.54 a	0.50 ± 0.05 a	1.87 ± 0.46 a	0.055 ± 0.008 a	13.96 ± 1.00 a
Two-way ANOVA (*p*-value)					
Biochar	*p* = 0.016	*p* = 0.003	*p* = 0.013	*p* = 0.026	*p* > 0.05
Bacteria	*p* = 0.005	*p* = 0.009	*p* = 0.004	*p* = 0.025	*p* = 0.013
Biochar × Bacteria	*p* = 0.045	*p* > 0.05	*p* > 0.05	*p* > 0.05	*p* > 0.05

Mean ± standard deviation, *n* = 3. Different lowercase letters in the column indicate significant differences among treatments by one-way ANOVA (LSD tests, *p* < 0.05).

**Table 2:** Analysis of stress tolerance of *Brassica chinensis* L.

Treatment	Chlorophyll (mg/g)	Root Activity (μg/min/g)	MDA (nmol/g)	Pro (μg/g)	SOD (U/g)	POD (U/g)
CK	34.48 ± 1.76 a	0.43 ± 0.06 ab	8.44 ± 1.11 a	63.71 ± 17.90 a	2012.62 ± 363.51 a	12,316.67 ± 1333.57 a
SS	37.37 ± 1.83 a	0.38 ± 0.02 b	9.03 ± 0.58 a	69.88 ± 1.13 a	2063.04 ± 225.22 a	13,192.00 ± 1025.01 a
JF	36.57 ± 4.00 a	0.49 ± 0.14 ab	8.06 ± 1.54 a	68.88 ± 16.92 a	2289.17 ± 194.49 a	13,034.67 ± 1083.85 a
JS	38.66 ± 1.22 a	0.57 ± 0.12 a	7.65 ± 0.98 a	71.36 ± 22.84 a	2356.81 ± 90.26 a	14,281.33 ± 2765.43 a
Two-way ANOVA (*p*-value)						
Biochar	*p* > 0.05	*p* > 0.05	*p* > 0.05	*p* > 0.05	*p* > 0.05	*p* > 0.05
Bacteria	*p* > 0.05	*p* > 0.05	*p* > 0.05	*p* > 0.05	*p* > 0.05	*p* > 0.05
Biochar × Bacteria	*p* > 0.05	*p* > 0.05	*p* > 0.05	*p* > 0.05	*p* > 0.05	*p* > 0.05

Mean ± standard deviation, *n* = 3. MDA, Pro, SOD, and POD refer to malondialdehyde, proline, superoxide dismutase, and peroxidase, respectively. Different lowercase letters in the column indicate significant differences among treatments by one-way ANOVA (LSD tests, *p* < 0.05).

**Table 3:** Analysis of soil physicochemical properties and enzyme activities.

Treatment	pH	Salt Content (g/kg)	EC (mS/cm)	SOM (%)	TN (%)	TP (%)	TK (%)	S-CAT (μmol/d/g)	S-UE (μg/d/g)	S-SC (mg/d/g)
CK	7.87 ± 0.01 a	1.22 ± 0.24 a	0.24 ± 0.021 abc	1.69 ± 0.21 a	0.17 ± 0.003 a	0.29 ± 0.009 b	1.92 ± 0.04 a	61.48 ± 1.92 b	623.71 ± 25.51 a	79.48 ± 21.77 a
SS	7.54 ± 0.06 b	1.21 ± 0.10 a	0.28 ± 0.004 a	2.10 ± 0.41 a	0.17 ± 0.029 a	0.32 ± 0.010 a	1.88 ± 0.03 a	66.29 ± 0.41 a	559.44 ± 49.57 a	116.44 ± 29.90 a
JF	7.52 ± 0.10 b	1.03 ± 0.01 ab	0.19 ± 0.002 b	2.17 ± 0.23 a	0.17 ± 0.015 a	0.30 ± 0.004 b	1.93 ± 0.03 a	62.71 ± 2.43 b	634.48 ± 93.66 a	72.37 ± 5.65 a
JS	7.60 ± 0.04 b	0.83 ± 0.07 b	0.19 ± 0.002 c	2.40 ± 0.68 a	0.18 ± 0.010 a	0.30 ± 0.007 b	1.93 ± 0.06 a	66.91 ± 1.61 a	632.34 ± 110.35 a	93.35 ± 27.14 a
Two-way ANOVA (*p*-value)										
Biochar	*p* = 0.003	*p* = 0.007	*p* < 0.001	*p* > 0.05	*p* > 0.05	*p* >0.05	*p* > 0.05	*p* > 0.05	*p* > 0.05	*p* > 0.05
Bacteria	*p* = 0.008	*p* > 0.05	*p* > 0.05	*p* > 0.05	*p* > 0.05	*p* = 0.007	*p* > 0.05	*p* = 0.002	*p* > 0.05	*p* > 0.05
Biochar × Bacteria	*p* < 0.001	*p* > 0.05	*p* = 0.010	*p* > 0.05	*p* > 0.05	*p* = 0.050	*p* > 0.05	*p* > 0.05	*p* > 0.05	*p* > 0.05

Mean ± standard deviation, *n* = 3. S-CAT, S-UE, and S-SC refer to soil catalase, urease, and sucrase, respectively. Different lowercase letters in the column indicate significant differences among treatments (LSD tests following one-way ANOVA for pH, Salt, TP, TK, S-CAT, S-UE, and S-SC; Games–Howell tests following Welch’s ANOVA for EC, SOM, and TN; *p* < 0.05).

**Table 4:** Analysis of soil quality index and contribution rate.

Treatment	Soil Quality Index	Improvement Rate (%)	Shapley Value (φ)	Contribution Rate (%)
CK	0.313 ± 0.065	-	-	-
SS	0.444 ± 0.117	41.99	0.134	44.33
JF	0.479 ± 0.098	52.90	0.168	55.67
JS	0.614 ± 0.077	96.23	+0.004	-

**Table 5:** The α diversity analysis of soil microbial communities.

Treatment	Chao1	Shannon	Simpson	Pielou_e
CK	25,491.17 ± 174.45 c	6.79 ± 0.03 b	0.996 ± 0.000 a	0.682 ± 0.003 a
SS	26,697.37 ± 269.88 a	6.92 ± 0.05 a	0.996 ± 0.000 a	0.688 ± 0.005 a
JF	25,859.97 ± 257.30 bc	6.85 ± 0.12 ab	0.995 ± 0.001 a	0.675 ± 0.012 a
JS	26,323.33 ± 313.81 ab	6.83 ± 0.06 b	0.996 ± 0.001 a	0.685 ± 0.005 a
Two-way ANOVA (*p*-value)				
Biochar	*p* > 0.05	*p* > 0.05	*p* > 0.05	*p* > 0.05
Bacteria	*p* < 0.001	*p* > 0.05	*p* > 0.05	*p* > 0.05
Biochar × Bacteria	*p* = 0.038	*p* > 0.05	*p* > 0.05	*p* > 0.05

Mean ± standard deviation, *n* = 3. Different lowercase letters in the column indicate significant differences among treatments by one-way ANOVA (LSD tests, *p* < 0.05).

**Table 6:** Background physicochemical properties of the saline–alkali soil.

pH	SOM (%)	TN (%)	TP (%)	TK (%)	EC (mS/cm)	Salt Content (g/kg)
7.94	2.07	0.08	0.24	1.79	0.46	2.88

SOM: soil organic matter; TN: total nitrogen; TP: total phosphorus; TK: total potassium; EC: Electrical conductivity.

## Data Availability

The original contributions presented in this study are included in the article/Supplementary Materials. Further inquiries can be directed to the corresponding authors.

## Supplementary Materials

The following supporting information can be downloaded at: https://www.mdpi.com/article/10.3390/plants15192987/s1, Figure S1: Correlation analysis of dominant taxa with soil properties and enzyme activity at phylum (top 10, a) and genus level (top 15, b). * *p* < 0.05, ** *p* < 0.01, with *n* = 3 replicates. *p*-values were adjusted using the Benjamini–Hochberg FDR correction; no correlations remained significant after adjustment (all adjusted *p* > 0.05); Figure S2: NMDS ordination of microbial β-diversity. (a) Order-level taxonomic composition (ANOSIM R = 0.330, *p* = 0.013; stress = 0.0949). (b) KEGG L2 functional profiles (ANOSIM R = 0.364, *p* = 0.028; stress = 0.0253). Table S1: Changes of the physicochemical properties of chicken manure biochar; Table S2: Changes of element contents of chicken manure biochar; Table S3: Immobilization of biochar-based agent with different ratios of MCMB; Table S4: Five retained Principal components; Table S5: Principal component-derived indicator weights via the communality method.
