# Supplementary material for: Effects of Modified Chicken Manure Biochar-Based Bacillus velezensis Agent on Vegetable Growth, Saline–Alkali Soil Properties, and Microbial Communities
Source: Plants (Basel). 2026 Sep 30;15(19):2987. doi: 10.3390/plants15192987 (PMC13667059; doi:10.3390/plants15192987)
Supplement: Supplementary file 1 [file plants-15-02987-s001.zip › plants-4479868-supplementary.pdf]

Determination of biochar

Biochar samples were mixed with CO_2-free water at a 1:15 (w/v) ratio, and the pH of the resulting suspension was measured with a pH meter (SevenCompact, Mettler Toledo, Greifensee, Switzerland). The specific surface area was quantified by the Brunauer–Emmett–Teller (BET) method (3Flex, Micromeritics, Norcross, GA, USA), in which the amount of nitrogen adsorbed onto the biochar surface at the temperature of liquid nitrogen (77 K) is recorded over a range of relative pressures and fitted to the multi-point BET equation. Pore volume, pore diameter, and porosity were tested with a mercury intrusion porosimeter (Autopore 9600, Micromeritics, Norcross, GA, USA). The pressure required for intrusion is inversely related to the pore size according to the Washburn equation, which allows the pore network from mesopores to macropores to be resolved in detail. The contents of carbon (C), hydrogen (H), nitrogen (N), and sulfur (S) were determined by an organic elemental analyzer (Unicube, Elementar, Langenselbold, Germany), in which the sample is combusted at high temperature and the evolved gases are separated and quantified. The contents of phosphorus (P), potassium (K), calcium (Ca), sodium (Na), and magnesium (Mg) were measured via inductively coupled plasma optical emission spectrometry/mass spectrometry (ICP-OES/MS) (iCAP, Thermo Fisher, Waltham, MA, USA) after acid digestion of the samples, yielding the mineral nutrient composition that is directly relevant to the agronomic value of the biochar.

Surface functional groups were characterized using Fourier transform infrared spectroscopy (FTIR) (Nicolet iS5, Thermo Fisher, Madison, WI, USA), which identifies characteristic oxygen-containing and aromatic moieties, such as hydroxyl, carbonyl, and aromatic C=C groups, on the basis of their specific infrared absorption bands and thereby reveals the chemical nature of the biochar surface. Finally, the biochar micromorphology, including particle shape, surface texture, and pore structure was observed via scanning electron microscopy (SEM) (Sigma 300, Carl Zeiss Microscopy, Jena, Germany), which scans the sample surface with a focused electron beam and images the topography through the detection of secondary electrons, providing direct visual evidence of the porous architecture of the biochar.

Table S1. Changes of the physicochemical properties of chicken manure biochar.

**Biochar type**	**Surface area (m^2/g)**	**Average pore (size/nm)**	**Pore volume (mL/g)**	**Micropore volume (cm^3/g)**	**Porosity (%)**
Raw chicken manure biochar	6.47	1716.30	0.62	0.000157	49.68
MCMB	11.18	1260.49	0.60	0.001738	53.81

MCMB refers to chicken manure biochar with acid modification.

Table S2. Changes of element contents of chicken manure biochar.

**Biochar type**	**C (%)**	**H (%)**	**N (%)**	**S (%)**	**P (%)**	**K (%)**	**Ca (%)**	**Na (%)**	**Mg (%)**
Raw chicken manure biochar	10.76	0.82	0.93	0.29	1.373	1.540	6.521	0.319	0.959
MCMB	9.59	1.05	1.24	2.46	0.040	0.140	6.517	0.0451	0.189

MCMB refers to chicken manure biochar with acid modification.

Table S3. Immobilization of biochar-based agent with different ratios of MCMB.

Mixing ratio	Total load of bacteria (10^{10} CFU)	Load of bacteria per gram (10^9 CFU/g)	Loading rate (%)
5%	2.58	5.16	40.95
10%	3.87	3.87	61.43
15%	4.18	2.79	66.34

Analysis of soil quality index

A total of 10 soil indicators were included in the SQI calculation. Principal components were retained until the cumulative variance contribution reached ≥ 85%, resulting in 5 retained PCs (Table S4). Within each retained PC, the indicator with the highest absolute factor loading was identified. Additionally, indicators with strong pairwise correlations ($|r| > 0.70$) were screened to avoid redundancy, retaining the one with the higher communality. No indicator pairs exceeded the threshold. Consequently, all 10 indicators were retained in the final minimum data set (MDS) and contributed to the SQI (Table S5).

If the soil indicator is positively correlated with soil quality, the "more-is-better" type indicator scoring (1) is used. Conversely, the "less-is-better" type indicator scoring (2) is used. The "optimum-is-better" type indicator scoring (3) is used for soil pH. The calculation formulas are:

$$Si = \frac{X - Xmin}{Xmax - Xmin} \quad (1)$$

$$Si = \frac{Xmax - X}{Xmax - Xmin} \quad (2)$$

$$Si = \frac{Xmax - X}{Xmax - Xopt} \quad (3)$$

where, Si is the score of each soil indicator; X is the measured value; $Xmax$ is the maximum value; $Xmin$ is the minimum value; $Xopt$ for soil pH is 7.0 in this paper.

Weights (Wi) were derived from PCA results as Eq. (4),

$$Wi = \frac{Vi}{\sum Vi} \quad (4)$$

where Vi is the proportion of variance explained by the PC containing indicator i.

Table S4. Five retained Principal components.

Principal components	Eigenvalue	Contribution rate of variance	Cumulative variance
PC1	3.323	30.46%	30.46%
PC2	2.918	26.75%	57.21%
PC3	1.423	13.05%	70.25%
PC4	1.043	9.56%	79.81%
PC5	0.764	7.00%	86.81%

Table S5. Principal component–derived indicator weights via the communality method.

Indicator	Communality (h^2)	Weight (Wi)	Weight Proporation
TN	1.020	0.1077	10.77%
S-UE	1.014	0.1071	10.71%
EC	1.006	0.1063	10.63%

SOM	0.977	0.1032	10.32%
S-CAT	0.977	0.1031	10.31%
TK	0.976	0.1031	10.31%
Salt content	0.908	0.0959	9.59%
TP	0.906	0.0957	9.57%
S-SC	0.901	0.0951	9.51%
pH	0.784	0.0828	8.28%

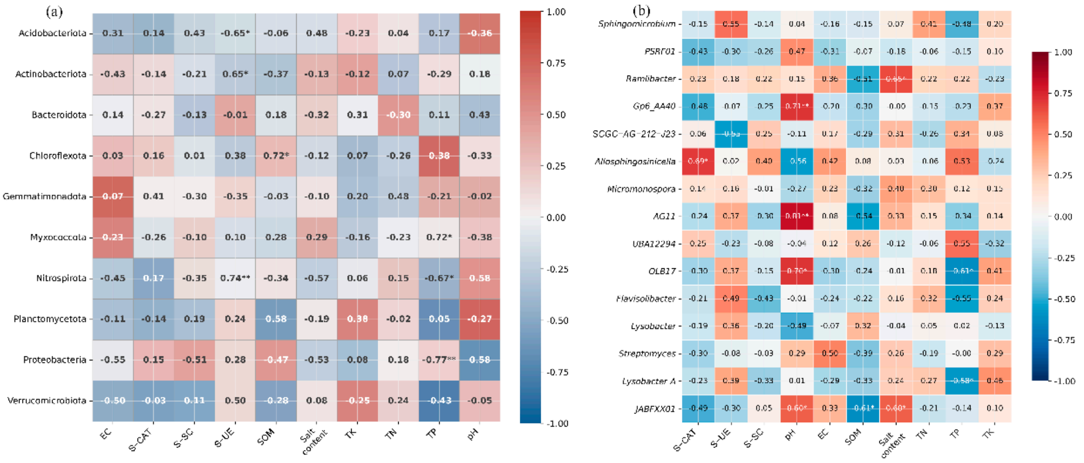

Figure S1. Correlation analysis of dominant taxa with soil properties and enzyme activity at phylum (top 10, **a**) and genus level (top 15, **b**). * p <0.05, ** p <0.01, with n = 3 replicates. p-values were adjusted using the Benjamini–Hochberg FDR correction; no correlations remained significant after adjustment (all adjusted p > 0.05)

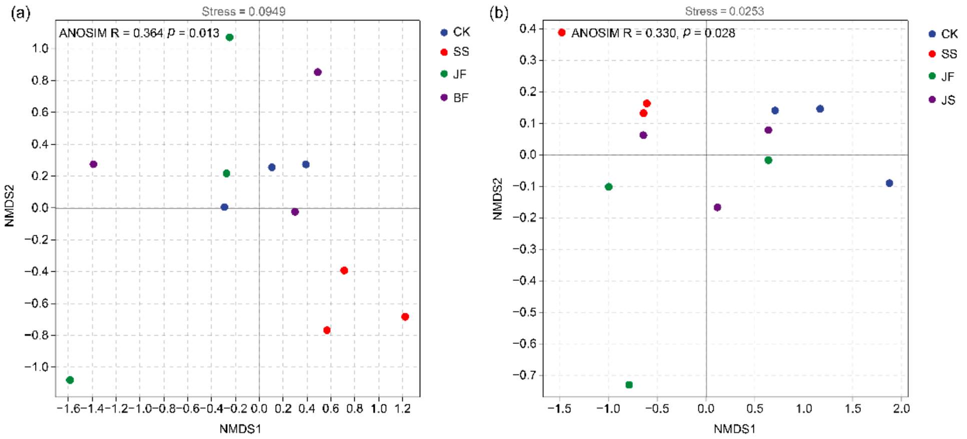

Figure S2. NMDS ordination of microbial β-diversity. (**a**) Order-level taxonomic composition (ANOSIM R = 0.330, p = 0.013; stress = 0.0949). (**b**) KEGG L2 functional profiles (ANOSIM R = 0.364, p = 0.028; stress = 0.0253).
